# *De Novo* Assembly, Gene Annotation, and Marker Discovery in Stored-Product Pest *Liposcelis entomophila* (Enderlein) Using Transcriptome Sequences

**DOI:** 10.1371/journal.pone.0080046

**Published:** 2013-11-14

**Authors:** Dan-Dan Wei, Er-Hu Chen, Tian-Bo Ding, Shi-Chun Chen, Wei Dou, Jin-Jun Wang

**Affiliations:** Key Laboratory of Entomology and Pest Control Engineering, College of Plant Protection, Southwest University, Chongqing, P. R. China; Auburn University, United States of America

## Abstract

**Background:**

As a major stored-product pest insect, *Liposcelis entomophila* has developed high levels of resistance to various insecticides in grain storage systems. However, the molecular mechanisms underlying resistance and environmental stress have not been characterized. To date, there is a lack of genomic information for this species. Therefore, studies aimed at profiling the *L. entomophila* transcriptome would provide a better understanding of the biological functions at the molecular levels.

**Methodology/Principal Findings:**

We applied Illumina sequencing technology to sequence the transcriptome of *L. entomophila*. A total of 54,406,328 clean reads were obtained and that *de novo* assembled into 54,220 unigenes, with an average length of 571 bp. Through a similarity search, 33,404 (61.61%) unigenes were matched to known proteins in the NCBI non-redundant (Nr) protein database. These unigenes were further functionally annotated with gene ontology (GO), cluster of orthologous groups of proteins (COG), and Kyoto Encyclopedia of Genes and Genomes (KEGG) databases. A large number of genes potentially involved in insecticide resistance were manually curated, including 68 putative cytochrome P450 genes, 37 putative glutathione *S*-transferase (GST) genes, 19 putative carboxyl/cholinesterase (CCE) genes, and other 126 transcripts to contain target site sequences or encoding detoxification genes representing eight types of resistance enzymes. Furthermore, to gain insight into the molecular basis of the *L. entomophila* toward thermal stresses, 25 heat shock protein (Hsp) genes were identified. In addition, 1,100 SSRs and 57,757 SNPs were detected and 231 pairs of SSR primes were designed for investigating the genetic diversity in future.

**Conclusions/Significance:**

We developed a comprehensive transcriptomic database for *L. entomophila*. These sequences and putative molecular markers would further promote our understanding of the molecular mechanisms underlying insecticide resistance or environmental stress, and will facilitate studies on population genetics for psocids, as well as providing useful information for functional genomic research in the future.

## Introduction

During the last two decades, several species of psocids from the genus *Liposcelis* (booklice) (Psocoptera: Liposcelididae) have emerged as serious pest insects of stored commodities in China and worldwide [1], [2]. As a new risk for global food security and safety, there is a growing awareness of the important pest status of psocids due to their economic impact on stored grains, contamination of food, and their role in the transmission of harmful microorganisms [3]–[5]. As a representation of psocids, *Liposcelis entomophila* (Enderlein) was the most commonly occurring species and had been proved difficult to control because they do not respond to management tactics that have been developed for other stored-product pests [6]. Recent studies have shown *L. entomophila* to be quite tolerant to some of the currently used insecticides, such as fenitrothion, chlorpyrifos-methyl, deltamethrin, bioresmethrin, and carbaryl [7]. Moreover, this species was also tolerant to permethrin and pirimiphos-methyl applied as structural treatments and to spinosad applied as a grain protectant [8], [9]. However, the regulatory molecular mechanisms of insecticides resistance in this species remain largely unknown. In insects, insecticide resistance commonly arises by two main mechanisms: metabolism or sequestration of insecticides or changes in the sensitivities of the target proteins. The first mechanism involves enhanced metabolism enzymes [10], such as carboxyl/cholinesterases (CCEs), glutathione *S*-transferases (GSTs), and cytochrome P450 (P450s). The other mechanism occurs through target mutation by reducing binding of the insecticide to its target [11], e.g. ryanodine receptors (RyRs) for phthalic acid diamides and anthranilic diamides, voltage-gated sodium channels (VGSCs) for pyrethroid, acetylcholinesterase (AChE) for organophosphates and carbamates, the GABA receptors for avermectins.

The small, soft-bodied and wingless *L. entomophila* showed a considerable degree of morphological and physiological variation both between and within clones [12]. Based on analysis of mitochondrial and nuclear DNA sequences of *L. entomophila*, it revealed that this psocid displayed high genetic diversity and widespread population genetic differentiation, and it implied that *L. entomophila* displayed considerable adaptability to deal with local or temporary situations [1]. Meanwhile, carbon dioxide-enriched atmospheres and heat treatments were also ineffective for managing this pest [13]. Recent study suggested that greater heat tolerance in *L. entomophila* might lead to its more common occurrence in grain stored in warmer regions of the world [14]. Thus, the greater heat tolerance and higher heterogeneity of response to heat shock in *L. entomophila* might facilitate its development of thermal tolerance or resistance to heat treatments in the grain storage facilities [14]. At present, a few studies have been conducted mainly focus on the ecology, molecular identification, and physiology and biochemistry of *L. entomophila* [4], [15]–[17]. However, discovery of the underlying biochemical and physiological mechanisms of *L. entomophila* to insecticides resistance and adaptability to adverse environments remains challenging. Indeed, existing genetic resources for *L. entomophila* are scarce, with less than 160 entries in the public database (NCBI) until on 6th August 2013 (available at http://www.ncbi.nlm.nih.gov). Among these 160 sequences, only 45 sequences are protein-coding genes, including two acetylcholinesterase, three cytochrome oxidase subunit I (COI) genes, one beta-actin, and 39 haplotypes of cytochrome b gene. Therefore, the lack of molecular and genomics data for this species has hampered characterization of the molecular mechanisms underlying insecticide resistance and environmental stress.

Fortunately, new high-throughput sequencing technologies referred to as ‘Next-generation Sequencing’, such as Solexa/Illumina, SOLID/ABI, 454/Roche platform, enable the generation of large amounts of sequence data on a far smaller timescale [18]. It is well known that the generation of large-scale expressed sequences tags (ESTs) is a very useful approach to describe the gene expression profile and sequences of mRNA from a specific organism and stage (especially in non-model species). ESTs represent a valuable sequences resource for research, due to they can provide comprehensive information regarding the transcriptome [19]. They play significant roles in functional genomics research for discovery of interesting genes, developing molecular markers, allowing large-scale expression analysis and improving genome annotation [20]–[23]. By generating sufficiently sequences reads, Illumina sequencing technology makes it possible to assemble high quality of transcriptomic sequences database. So far, a large number of insects have been successfully performed for transcriptome analyses by *de novo* assembly of Illumina sequences [19], [24]–[27]. In the present work, we used Illumina sequencing technology to generate a substantial EST dataset of *L. entomophila*, a species that has been the focus of extensive study but has lacked genomic resources and then characterized genes encoding detoxification enzymes, insecticide target proteins, and environmental stress related genes as well as putative molecular markers. This study dramatically provides a foundation and increases the significant promise for further functional genomics studies of *L. entomophila* and other *Liposcelis* species.

## Materials and Methods

### Ethics Statement

No specific permits were required for the insects collected in this study. No specific permissions were required for these locations/activities which the insect specimens were collected. We confirm that these locations are not privately-owned or protected in any way and the species collections did not involve endangered or protected species.

### Insect samples

*Liposcelis entomophila* were collected at grain storage facilities from three locations in three provinces (Chongqing, Hubei, and Hainan) of China from 2008–2010. The insects were cultured in the laboratory with artificial diet consisting of whole wheat flour, skim milk, and yeast powder (10∶1∶1) in an incubator at 27±0.5°C and 75–80% relative humidity and a scotoperiod of 24 h. We initiated the insect culture with the uniform eggs to obtain the insects in different but uniform developmental stages including eggs, nymphs, and adults (male∶female = 1∶1). The experiments were replicated three times (each time for one population) for each developmental stage, and each stage was taken as one sample for total RNA extraction, respectively.

### RNA isolation, library construction and Illumina sequencing

Total RNA for each sample was isolated using the RNeasy Plus Micro Kit (Qiagen, Hilden, Germany) according to the manufacturer's instructions. For each sample, RNA quantities were assessed at an absorbance ratio of OD_260/280_ with NanoVue spectrophotometer (GE Healthcare Bio-Science, Uppsala, Sweden). The purity and degradation of RNA was also checked on 1% agarose gel electrophoresis. The mixture of RNA from all developmental stages of three populations with equal ratio pooled as one sample for constructing cDNA library.

Oligo (dT) magnetic beads were used to isolate poly (A) mRNA, which was then fragmented to 200–700 bp. These short fragments were used for the first-strand cDNAs synthesis using random hexamer primers. The cDNA synthesis of the second-strand was performed using DNA polymerase I (New England BioLabs, Ipswich, MA) and RNase H (Invitrogen, Carlsbad, CA). These cDNA fragments were purified and resolved with ethidium bromide buffer for end reparation and single nucleotide A (adenine) addition and then ligated to adaptors. These ligation products were subjected to agarose gel electrophoresis and the suitable fragments were amplified by PCR to create the final cDNA library. The cDNA library was sequenced on the channels of an Illumina HiSeq™ 2000 instrument for four gigabase in-depth, which was used to obtain more detailed information about expressed genes.

### *De novo* assembly of sequencing reads and bioinformatics analysis

Raw reads produced from sequencing machines were filtered to remove reads with adaptors, low-quality sequences (reads with unknown sequences ‘N’), reads with more than 20% low quality (base quality <10) bases and empty sequences (sequences with only adaptor but no reads). The clean read data has been deposited in the NIH Short Read Archive (SRA) database (Accession No. SRP028706). The *de novo* assembly of the clean reads was carried out with short reads assembling program Trinity [28]. Briefly, clean reads with a certain overlap length were combined to form longer contiguous sequences (contigs) and mapped back onto the contigs. The contigs were processed by Trinity and could not be extended on either end, and the result sequences were called unigenes. Due to multiple samples from the same species were sequenced, these unigenes assembly should be taken into further process of sequences splicing and redundancy removing. Thus, the unigenes were divided into two classes. One was clusters, which were several unigenes that had more than 70% similarity between them and the other was singletons.

All assembled unigenes were determined by BLASTx against NCBI non-redundant (Nr) protein database, Swiss-Prot, the Kyoto Encyclopedia of Genes and Genomes (KEGG) database and Cluster of Orthologous Groups (COG). The *E*-value cut-off was set at 1.0E^−5^. Genes were tentatively identified according to the best hits against known sequences. If the alignment results of different databases conflicted with each other, the priority order of Nr, Swiss-Prot, KEGG, and COG were followed when deciding sequence direction of unigenes. Sequences with BLASTx hits were annotated according to Gene Ontology (GO) terms using Blast2GO software [29].

### Manual curation of interesting gene and phylogenetic analysis

Interesting sequences that related to insecticide detoxification, the target sites of the most important insecticides, as well as unigenes that corresponded to the thermal stress were searched by BLAST results against the Nr database with a cut-off *E*-value <1.0E^−5^. The unigenes found in the same BLAST results or with high homology to one another were eliminated selectively as allelic variants or as different parts of the same gene. The coding region was determined by the ORF finder (http://www.ncbi.nlm.nih.gov/gorf/gorf.html) and further checked by protein BLAST results. Statistics of the number of genes from major detoxification families were investigated across different insect species. Some of these insects have a close relationship with psocids (the hemipteroid assemblage, include insects from Hemiptera and Phthiraptera order) or those model insects which have the detailed information of detoxification genes through genomic or transcriptomic sequencing. The family genes of P450s, GSTs, CCEs, and Hsps were aligned at the amino acid level using the default settings in ClustalW (as implemented in MEGA 5 [30]) and then to construct consensus phylogenetic trees using Neighbour-Joining (NJ) method to make a prediction of their classification. NJ phylogenetic trees were estimated using the bootstrap test with 1,000 replicates.

### Molecular marker detection

All the unigenes were used to screen for microsatellites. The screening was performed in MicroSAtellite (MISA) (http://pgrc.ipk-gatersleben.de/misa/) [31], which could identify the microsatellites and simultaneously design the PCR primers using the inbuilt program PRIMER v. 3 [32]. The parameters were adjusted for identification of perfect di-, tri-, tetra-, penta-, and hexa-nucleotide motifs with a threshold of 6, 5, 5, 4, and 4 repeats, respectively. Only the length of the both ends on the unigene are more than 150 bp were adopted to design primers. The target amplicon size was set as 100–300 bp, with optimal annealing primer temperature of 58°C (range 55–62°C) and optimal primer length as 22 bp (range 18–26). The potential single nucleotide polymorphisms (SNPs) in *L. entomophila* Illumina data were predicted using the program SOAPsnp (http://seqanswers.com/wiki/SOAPsnp) [33]. The assembled unigene sequences were used as templates to BLAST the original sequencing reads.

## Results and Discussion

### Illumina sequencing and *de novo* assembly

To obtain a more comprehensive understanding of gene expression profile at different developmental stages in *L. entomophila*, a pooled cDNA sample from three populations, representing eggs, nymphs, and adults (females and males), was sequenced using the Illumina sequencing platform. Sequencing generated 62,698,056 raw reads, encompassing about 4.14 Gb sequencing data. After initial adaptor trimming and quality filtering, 54,406,328 clean reads were assembled into 106,774 contigs longer than 100 bp with a mean length of 317 bp (Table 1). Although the majority of the contigs were between 100 and 200 bp (60.73%), 19,407 (18.18%) were longer than 400 bp (Figure S1 B). Using paired-end joining and gap-filling methods, the contigs were further assembled into 54,220 unigenes (11,126 distinct clusters and 43,094 distinct singletons) with an average length of 571 bp (Table 1). Among the all total unigenes, 6,846 (12.63%) unigenes were longer than 1,000 bp, and 34,696 (63.99%) unigenes were less than 500 bp (Figure S1 A).

**Table 1:** Summary statistics for the Illumina sequencing from *Liposcelis entomophila* transcriptome.

Sequencing	
Total number of reads	62,698,056
Total number of clean reads	54,406,328
Total clean nucleotides (bp)	4,896,569,520
Q20 percentage (%)	97.09
N percentage (%)	0.01
GC percentage (%)	44.89
Number of contigs	106,774
Length of contigs (bp)	33,864,263
Mean length of contigs (bp)	317
Number of unigenes	54,220
Length of unigenes (bp)	30,953,334
Mean length of unigenes (bp)	571
Cluster of unigenes	11,126
Singleton of unigenes	43,094
Unigenes annotations against nr	33,404
Unigenes annotations against Swiss-Prot	25,637
Unigenes annotations against KEGG	23,068
Unigenes annotations against COG	11,773
Unigenes annotations against GO	19,355

The number of reads in the unigenes was also calculated, and it varied from 1 to 3,263,623 with a mean of 837. About 83.62% of the unigenes have the number of reads less than 500, whereas the unigenes with number of reads more than 5,000 only accounted for 2.13% of the total unigenes. The unigenes with reads more than two times of average (1,674 reads) were considered as highly expressed transcripts [34], and 3,320 highly expressed unigenes were found, accounting for 6.12% of the total unigenes. Here, the 13 unigenes with most abundant reads (>250,000 reads) represented four vitellogenin, two hexamerin, two elongation factor genes, one hypothetical protein, one actin, one glutathione *S*-transferase, one heat shock protein, and one chymotrypsin.

### Homology searches

To annotate *L. entomophila* unigenes, all of the assembled sequences were subjected to BLASTx similarity search against the NCBI non-redundant (Nr) protein database to determine their putative functions. A total of 33,404 unigenes (61.61%) had hit in the Nr protein database (Table 1). The remaining 20,816 sequences (38.39%) failed to acquire annotation information with cut-off *E*-value of 1.0E^−5^, suggesting that they may be specifically expressed in *L. entomophila* or due to these sequences correspond to untranslated regions (orphan UTRs) as well as errors in unigene assembly. Compared to the previous studies of insect transcriptome which were profiled by Illumina sequencing, the percentage of unigene sequences with homology to known proteins in our result is higher than that reported for oriental fruit fly (*Bactrocera dorsalis*) (55%) [27], the whitefly (*Bemisia tabaci*) (16.2%) [19], the brown planthopper (*Nilaparvata lugens*) (40%) [35], the diamondback moth (*Plutella xylostella*) (22.3%) [21], and the pine shoot beetle (*Tomicus yunnanensis*) (57.81%) [25]. Therefore, our results succeeded in annotating a significant proportion of putative genes in *L. entomophila* transcripts and given abundance genomic information for this pest.

**Figure 1:**
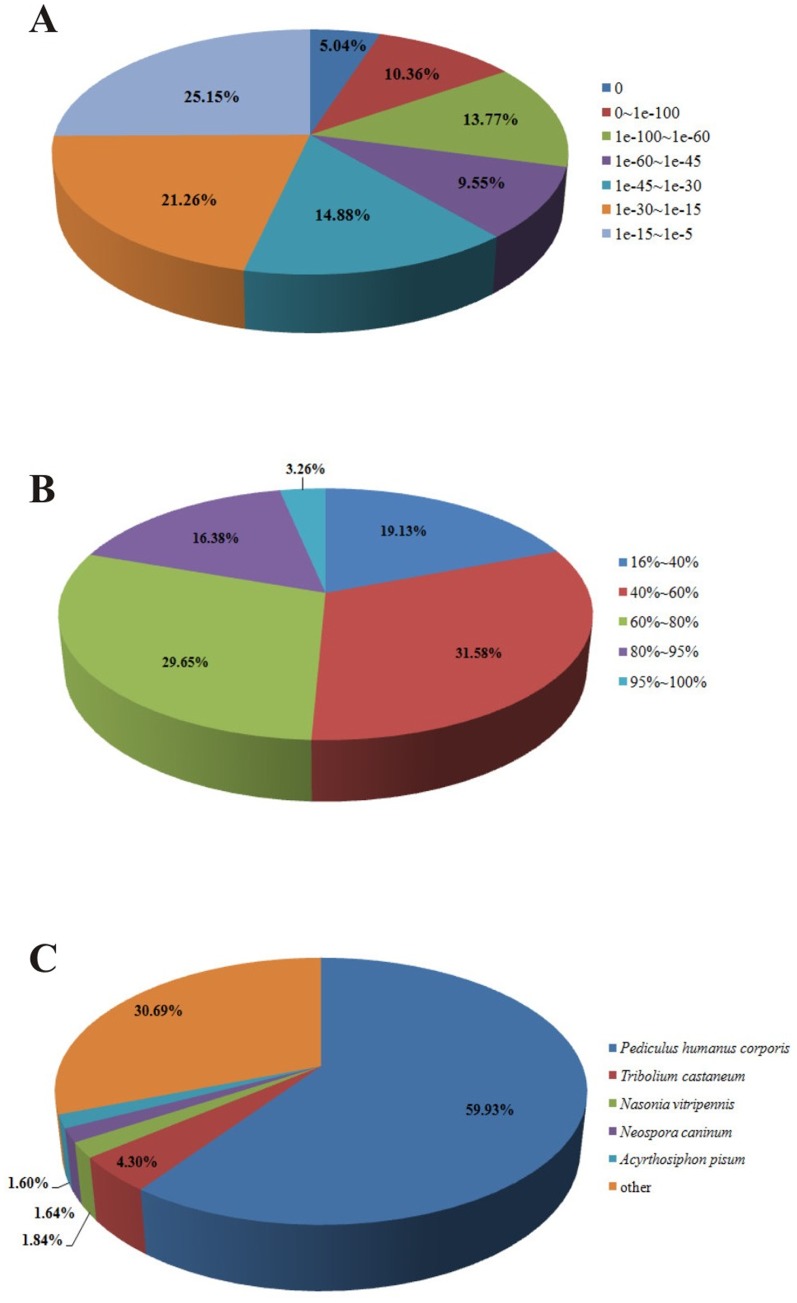
Homology analysis of unigenes for *Liposcelis entomophila*. **A**, E-value distribution of BLAST hits for each unigenes with a cut-off E-value of 1.0E^−5^; **B**, Similarity distribution of the top BLAST hits for each sequence; **C**, Species distribution.

The *E*-value distribution of these annotated unigenes showed that 38.71% of the mapped sequences had significant homology (<1.0E^−45^), whereas 61.29% of the homolog sequences ranged between 1.0E^−5^ to 1.0E^−50^ (Figure 1A). Further analysis of the BLAST data indicated that 6,561 (19.64%) annotated sequences had a similarity higher than 80%, and 26,842 (80.36%) of the hits had a similarity ranging from 16% to 80% (Figure 1B). According to the best hit in the Nr database, more than half of annotated unigenes (20,017 unigenes, 59.93%) had strong homology with human body louse (*Pediculus humanus*), while a relative low proportion (<40.70%) of them matched to other insects or organisms (Figure 1C). The species distribution was in our expectation. Recently, the genome of human body louse was fully sequenced [36], and *L. entomophila*, as the booklouse had a close relationship (the booklice in the genus *Liposcelis* are the sister-group to the species of Phthiraptera order) with the parasitic lice [37], [38].

### Function annotations

**Figure 2:**
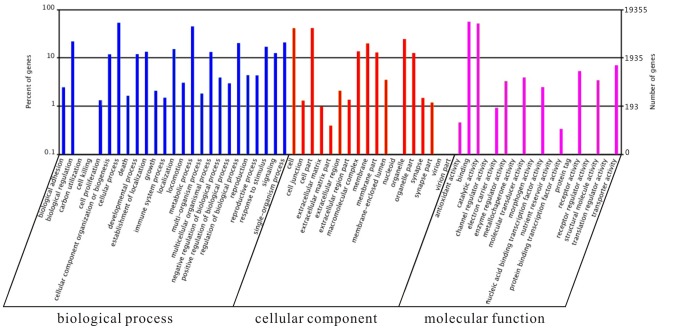
Gene Ontology (GO) terms for the transcriptomic sequences of *Liposcelis entomophila*.

GO is widely used to standardize representation of genes across species and provides a structured and controlled vocabulary for describing gene products [39]. A total of 19,355 unigenes were assigned at least one GO term based on their similarity to sequences with previously known functions (Table 1). These GO terms were summarized into the three main categories (biological process, cellular component, and molecular function) and 61 subcategories (Figure 2). Due to several unigenes were assigned to more than one GO term, the total number of GO terms (116,096) in our dataset was greater than the total number of unigenes. This result was consistent with other insect transcriptomes [40], and it implied that many putative genes in *L. entomophila* could be involved in a series of different physiological and biochemical processes. Among these unigene GO terms, the biological process (including 25 subcategories; 55,288, 47.62%) made up the majority, followed by cellular component (including 18 subcategories; 34,588, 29.79%) and molecular function (including 18 subcategories; 26,220, 22.58%) (Table S1). The distribution of GO terms within the ontology is consistent with many previous insect transcriptome studies [22], [25], [41]. Under the biological process, the major subcategories were cellular process (10,355, 8.92%) and metabolic process (8,663, 7.46%). For cellular component, the cell (8,011, 6.90%) and cell part (8,011, 6.90%) were most abundant. Within category of molecular function, binding (10,894, 9.38%) and catalytic activity (10,026, 8.64%) were highly represented. The high percentage of genes were assigned for binding, predominantly heat shock proteins (Hsps) and cellular processes such as proteolysis, carbohydrate metabolic processes and oxidation reduction utilization, suggesting that these proteins may play an important role under environment stress.

**Figure 3:**
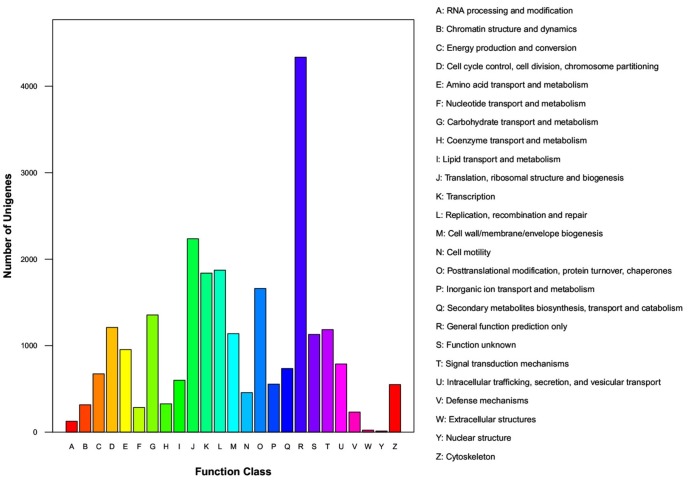
Clusters of orthologous group (COG) function classification of the *Liposcelis entomophila* transcriptome.

To further evaluate the effectiveness of the annotated unigenes, we aligned the unigenes to the COG database for functional prediction and classification. In total, 11,773 unigene sequences had a COG functional classification and these sequences were classified into 25 COG categories (Figure 3). Among these categories, ‘General function prediction’ represent the most common category (4,336, 36.83%), followed by ‘translation, ribosomal structure and biogenesis’ (2,236, 18.99%), and ‘replication, recombination and repair’ (1,873, 15.91%). ‘Extracellular structures’ (23, 0.2%) and ‘nuclear structure’ (13, 0.11%) were the two smallest groups.

In addition, KEGG pathway mapping based on enzyme commission numbers for assignments was carried out for all assembled unigenes, which is an alternative approach to categorize genes functions with emphasis on biochemical pathways [42]. In total, 23,068 *L. entomophila* unigenes were mapped on to 258 KEGG pathways. The most abundant pathways were involved in ‘metabolic pathways’ (3,132, 13.58%), followed by ‘Pathways in cancer’ (770, 3.34%) and ‘RNA transport’ (740, 3.21%) (Figure S2). The KEGG pathway analyses here are helpful for prediction potential genes and their functions at a whole transcriptome level and useful for further research of metabolic pathways, functions, and complicated biological behaviors of *L. entomophila* genes.

### Discovery of genes related to insecticides resistance

In the last two decades, the insect pest, *L. entomophila* has become a serious problem in grain storage systems, due to their economic impact on stored grains and developing high level of resistance to a wide range of synthetic insecticides [1], [8], [9], [43]. In this study, we have mined the current transcriptomic database to obtain genes potentially involved in insecticide resistance of *L. entomophila* (Table 2). The transcripts encoding genes involved in insecticide detoxification including P450 (266 unigenes), carboxylesterase (CarE, 100 unigenes), and GSTs (73 unigenes). The insecticide targets included acetylcholinesterase (AChE, 13 unigenes), nicotinic acetylcholine receptor (nAChRs, 28 unigenes), and the voltage-gated sodium channel (VGSC, 13 unigenes) (Table 2). As our understanding of the regulation of mechanisms underlying insecticide resistance increase, new chemicals, compounds or other strategies could be devised for the development of more efficient, eco-friendly and species-specific strategies for this pest control.

**Table 2:** Unigene sequences were potentially involved in insecticide resistance in *Liposcelis entomophila*.

Gene names	Number of unigenes hit with Nr database
Cytochrome P450	266
Glutathione *S*-transferases	73
Carboxylesterase	100
Acetylcholinesterase	13
Superoxide dismutase	6
Acetyl-CoA carboxylase	13
Catalase	1
Ryanodine receptor	20
Sodium channel	13
Chloride channel	36
Nicotinic acetylcholine receptor	28
GABA receptor	9

#### Cytochrome P450 (P450)

**Figure 4:**
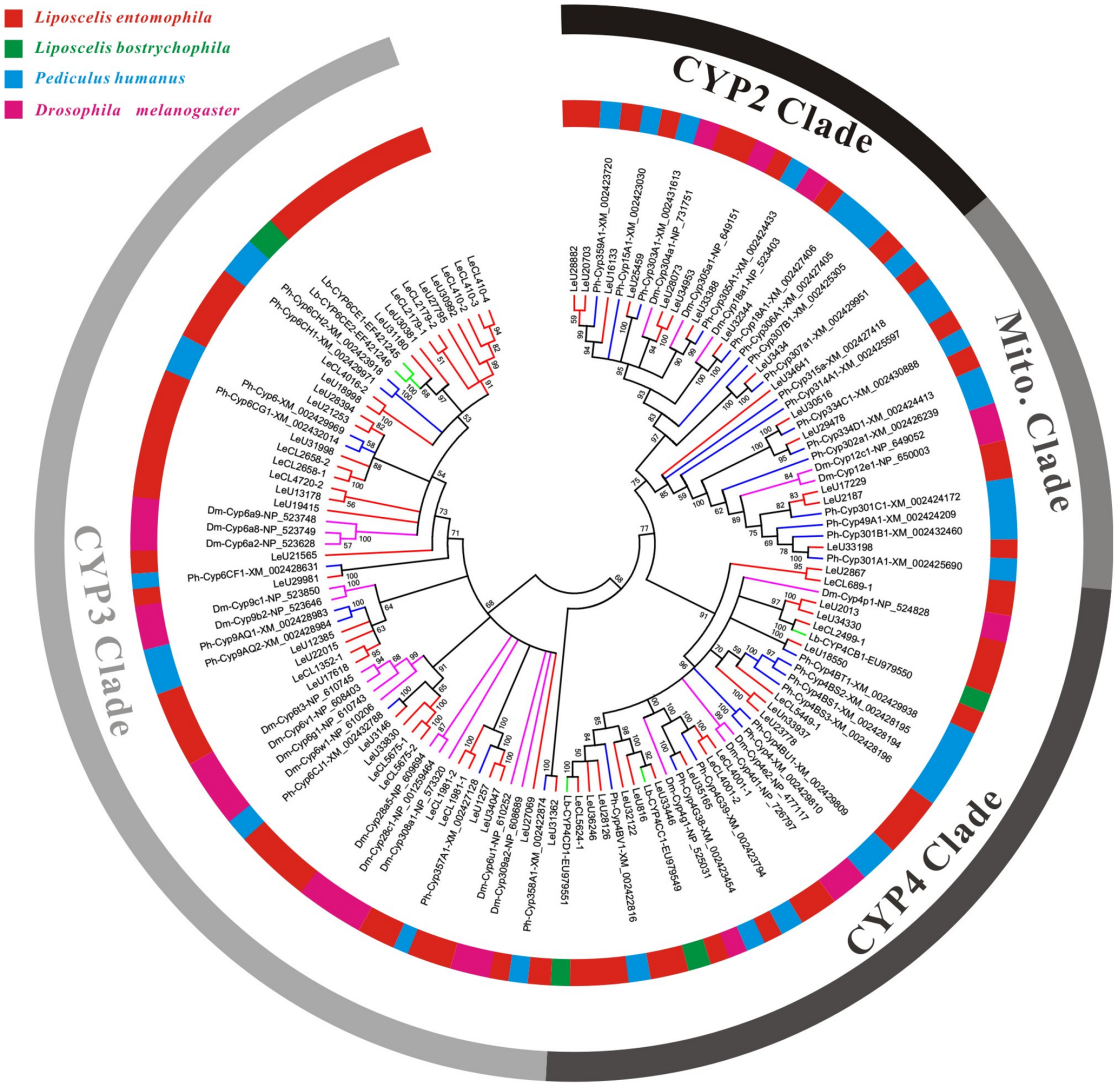
Phylogenetic analysis of putative cytochrome P450 genes in *Liposcelis entomophila* with other insects. Numbers above the branches show support for the phylogenies from amino acid sequences and only values above 50% are shown.

P450s (mixed function oxidases) are one of the largest families of genes with representatives in all living organisms and play a critical role in plant-insect interactions and insecticide/xenobiotic metabolism in pscoids and other insects [44], [45]. After manually removing the unigenes that have short open reading frames (ORFs) or have similarity with non-insect organisms, the remaining 68 putative P450 genes were identified (Table S2). The length of these P450 genes varied from 738 to1,626 bp with an average length of 1,124 bp. Based on phylogenetic analysis with other known insect P450s or the best BLAST hits against the Nr database, the *L. entomophila* P450 genes were assigned well to appropriate P450 clades and families (Figure 4). The majority of these P450 genes belonged to CYP3 clade (35/68) and CYP4 clade (18/38) compared to CYP2 clade (9/38) and Mitochondrial (6/38), which is in agreement with other insect systems [40], [46]–[48] (Figure 4 and Table 3).

**Table 3:** Class/clade and number distribution of the cytochrome P450s, glutathione *S*-transferases (GSTs) and carboxyl/cholinesterases (CCEs) in different insect species.

Enzymes/Class	Le	Ph	Ap	Mp	Tv	Tc	Am	Nv	Aa	Ag	Bo	Dm
**P450s**												
CYP2	9	8	10	3	3	8	8	7	12	10	2	6
CYP3	35	12	33	63	34	72	28	48	82	40	28	36
CYP4	18	9	32	48	13	45	4	30	57	46	17	32
Mitochondrial	6	8	8	1	7	9	6	7	9	9	13	11
Subtotal	**68**	**37**	**83**	**115**	**57**	**134**	**46**	**92**	**160**	**105**	**60**	**85**
**GSTs**												
Delta	17	4	10	8	9	3	1	5	8	12	8	11
Epsilon	0	0	0	0	1	19	0	0	8	8	12	14
Omega	1	1	0	0	0	4	1	2	1	1	3	5
Sigma	13	4	6	8	5	7	4	8	1	1	1	1
Theta	3	1	2	2	0	1	1	3	4	2	4	4
Zeta	1	1	0	0	1	1	1	1	1	1	3	2
Microsomal	2	2	2	2	1	0	2	0	0	3	0	1
Other	0	0	–	–	–	0	0	0	3	3	2	0
Subtotal	**37**	**13**	**22**	**20**	**17**	**35**	**10**	**19**	**26**	**31**	**33**	**38**
**CCEs**												
Dietary class												
A clade	5	3	5	5	11	0	8	8	0	0	0	0
B clade	0	0	0	0	0	14	0	5	22	14	0	2
C clade	0	0	0	0	1	12	0	0	0	2	7	11
Hormones and Pheromone												
D clade	5	0	0	0	0	2	1	4	0	0	2	3
E clade	3	1	18	12	6	7	3	11	2	4	1	3
F clade	1	0	0	0	0	2	0	2	6	4	0	2
G clade	0	0	0	0	0	0	1	0	6	4	0	0
H clade	3	1	1	0	1	1	0	1	7	9	2	4
Neurodevelopmental												
I clade	0	1	0	1	1	1	2	1	1	2	3	2
J clade	2	2	2	3	2	2	2	2	2	2	0	1
K clade	0	1	1	1	1	1	1	1	1	1	0	1
L clade	0	5	3	0	3	5	5	5	5	5	0	4
M clade	0	3	0	0	1	2	1	1	2	2	0	2
Subtotal	**19**	**17**	**29**	**22**	**27**	**49**	**24**	**41**	**54**	**51**	**15**	**35**

Le, *Liposcelis entomophila;* Ph, *Pediculus humanus;* Ap, *Acyrthosiphon pisum;* Mp, *Myzus persicae;* Tv, *Trialeurodes vaporariorum;* Tc, *Tribolium castaneum;* Am, *Apis mellifera;* Nv, *Nasonia vitripennis;* Aa, *Aedes aegypti;* Ag, *Anopheles gambiae;* Bo, *Bactrocera oleae;* Dm, *Drosophila melanogaster*. The dash ‘-’ indicated that the data were not available.

The most commonly members of the CYP3 clade (includes CYP3, CYP6, CYP9 members) in booklice and other insect species are due to their important functions against xeniobiotics and phytotoxins. According to Table 3, *L. entomophila* harbored a lot of P450 genes in CYP3 clade and this was quite different from its closest relatives, *P. humanus*, which lost most of this clade P450 genes. This implied that *L. entomophila* could quickly developed high level of insecticide resistance. However, when we screened these 68 P450 genes against the KEGG database annotations and found most of genes belonged to CYP4 and CYP3 did not map in KEGG database (Table S2). By family, ‘Insect hormone biosynthesis’ (ko00981) was the major pathway for CYP2, while ‘Drug metabolism - other enzymes’ (ko00983), ‘Drug metabolism - cytochrome P450’ (ko00982), ‘Metabolism of xenobiotics by cytochrome P450’ (ko00980), were the pathways for only two CYP3 genes (LeU17618 and LeU17618). In addition, these two CYP3 genes have the number of reads more than 1,674 (4,189 and 3,671) and both of them were considered as highly expressed transcripts. We propose that these two genes can expressed at a high level under the normal conditions for *L. entomophila*, and the other P450 genes might have basic biochemical function in metabolic pathways. Therefore, the future studies of *L. entomophila* P450 genes should pay more attentions to these two P450 genes, and which will advance our understanding of the role of P450s in psocids.

#### Glutathione S-transferases (GSTs)

**Figure 5:**
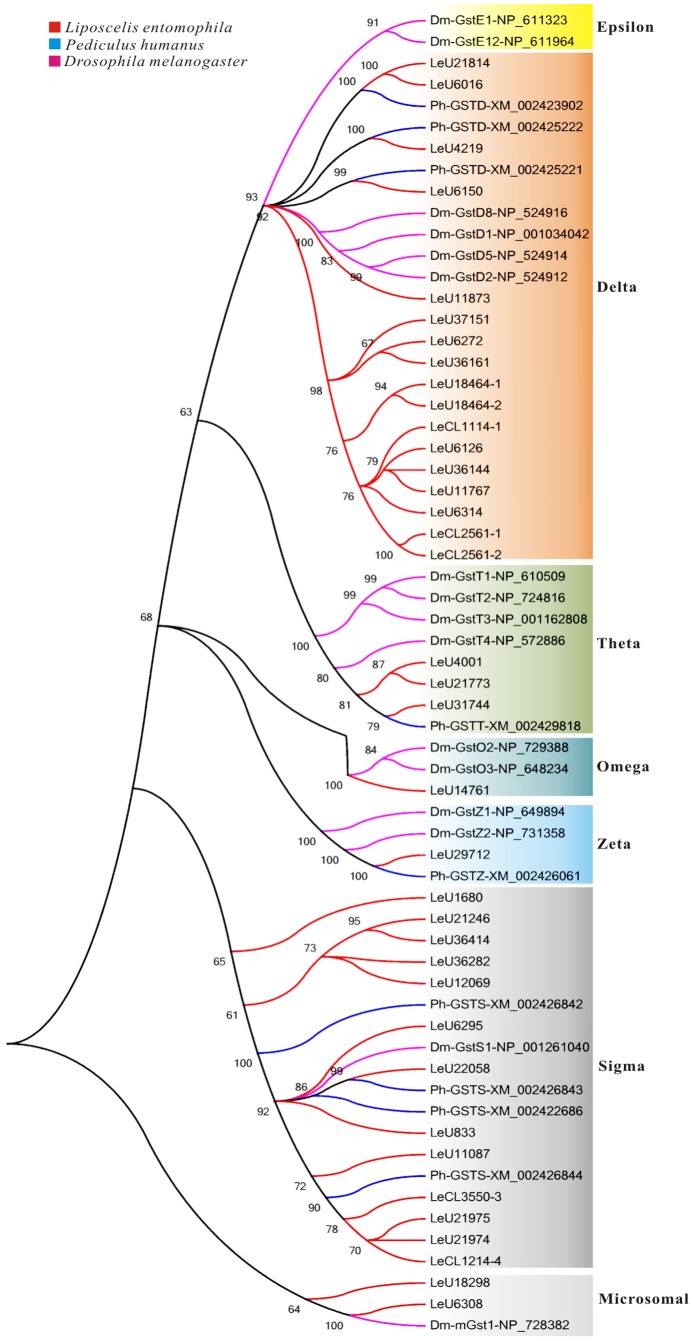
Phylogenetic analysis of putative glutathione *S*-transferase (GST) genes in *Liposcelis entomophila* with other insects. Numbers above the branches show support for the phylogenies from amino acid sequences and only values above 60% are shown.

A total of 73 GSTs transcripts were identified from Nr annotation of *L. entomophila* transcriptomic sequences, and 37 putative GSTs genes were manually curated after removing short unigenes. The GSTs super-family members are proved to be involved in the resistance to phytotoxins and insecticide [49]. Insect GSTs can be divided into seven classes (Delta, Epsilon, Omega, Sigma, Theta, Zeta, and Microsomal), and Delta and Epsilon are two unique classes to insects and are thought to contribute to the environmental variation [10]. In this study, 37 GSTs-specific genes were assigned to six classes, including Delta (17), Omega (1), Sigma (13), Theta (3), Zeta (1), and Microsomal (2) (Figure 5). No GSTs belonging to Epsilon class was identified, as is the cases for other insects such as *P. humanus*, *Acyrthosiphon pisum*, *Myzus persicae*, and *Apis mellifera* (Table 3). Furthermore, the number of GSTs genes from Sigma class in *L. entomophila* was far greater than other insects (Table 3), and this greater diversification of Sigma GSTs in *L. entomophila* and other insects was mainly a result of local gene duplication events [46]. On the other hand, evidence exists that insect Sigma GSTs play protective roles against oxidative stress [50] and metabolic roles in processing endogenous substrates as well as xenobiotics [51], [52]. Therefore, the Sigma GSTs in *L. entomophila* are likely to play general defensive roles in a broad sense and this corollary was further demonstrated by KEGG pathway analysis. In Sigma GSTs genes, the major pathway were ‘Prostate cancer’ (ko05215), ‘Glutathione metabolism’ (ko00480), ‘Drug metabolism - cytochrome P450’ (ko00982), ‘Metabolism of xenobiotics by cytochrome P450’ (ko00980) and ‘Arachidonic acid metabolism’ (ko00590) (Table S3). In fact, the pathway ‘Prostate cancer’ (ko05215), ‘Glutathione metabolism’ (ko00480), ‘Drug metabolism - cytochrome P450’ (ko00982), ‘Metabolism of xenobiotics by cytochrome P450’ (ko00980) were also the majority of pathways for other class GSTs (Delta, Theta and Omega) (Table S3). Meanwhile, the relatively similar distribution of genes in classes of GSTs (Delta, Theta, Omega and Zeta) across different insect species (Table 3), suggests that they have more common conserved functions in physiological processes, including the metabolism of endogenous substrate and cellular defense against oxidative stress. Moreover, it is worth to note that almost all of GSTs genes had the pathways ‘Drug metabolism - cytochrome P450’ and ‘Metabolism of xenobiotics by cytochrome P450’, which suggested their involvement in xenobiotics detoxification. Intriguingly, these two pathways were not identified in most of P450 genes, and this implied that GSTs might play more important defensive role against environmental stress than P450s in *L. entomophila*.

#### Carboxyl/cholinesterases (CCEs)

**Figure 6:**
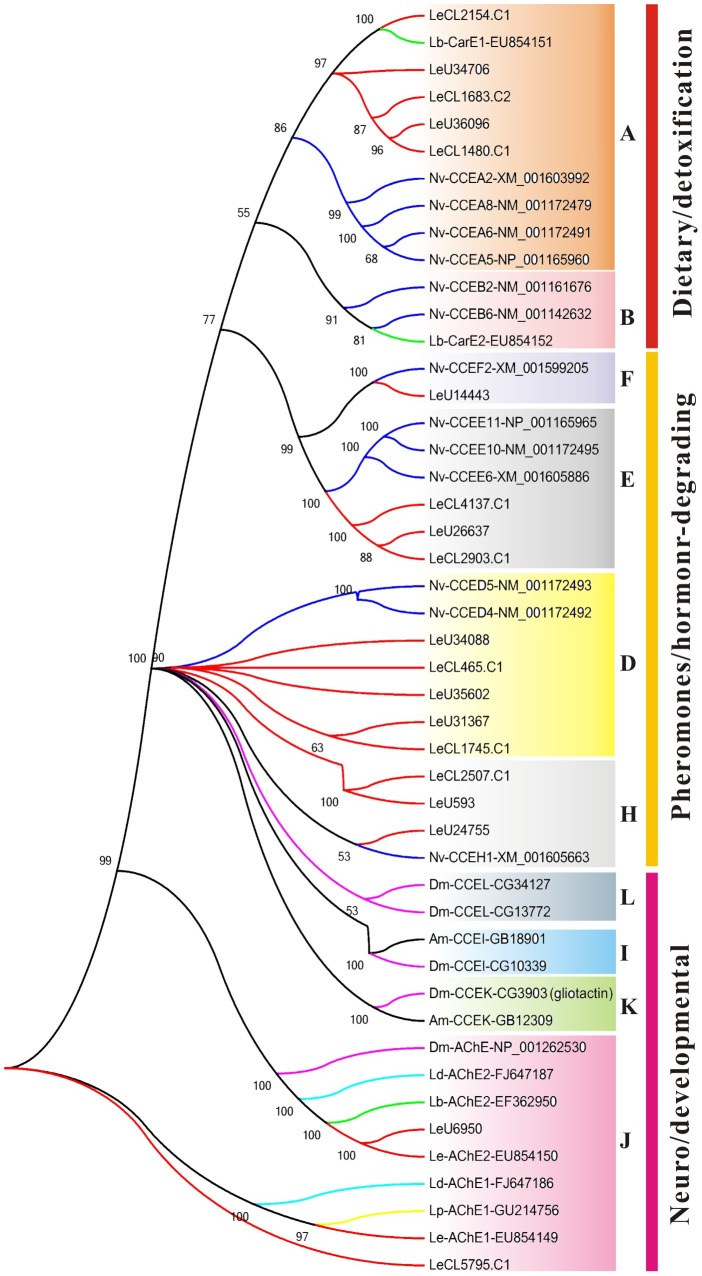
Phylogenetic analysis of putative carboxyl/cholinesterase (CCE) genes in *Liposcelis entomophila* with other insects. Numbers above the branches show support for the phylogenies from amino acid sequences and only values above 50% are shown. Le, *Liposcelis entomophila*; Lb, *Liposcelis bostrychophila*; Nv, *Nasonia vitripenni*s; Dm, *Drosophila melanogaster*; Ld, *Liposcelis decolor*; Lp, *Liposcelis paeta*.

In insects, CCEs can be categorized into three classes including 13 clades: the first class (clades A-C) contains primarily intracellular esterases with dietary detoxification functions; the second class (clades D-H) contains secreted and catalytically active esterases, including juvenile hormone esterases (JHEs); and the third class (clades I-M) contains esterases with neuro/developmental function, including acetylcholinesterases [53]. CCEs have shown to be involved in the detoxification of insecticides as well as metabolism of plant derived allelochemicals [54]. In *L. entomophila* transcriptomic database, 19 putative CCE genes were manually identified after removing short sequences or allelic variants. These CCEs genes have a length of range from 1,116-1,914 bp and with an average of 1,427 bp (Table S4). Based on phylogenetic analysis with other known insect CCEs, these CCEs were assigned to six clades (5 belong to A clade, 5 to D clade, 3 to E clade, 1 to F clade, 3 to H clade and 2 to J clade) (Figure 6). The number of CCEs from *L. entomophila* is less than most of other insect CCEs and no CCEs were identified belong to B, C, G, I, K-M clades (Table 3). Though the number of CCEs in the *L. entomophila* transcriptome is within the range of CCEs identified in other insect species (15–54) (Table 3), additional CCEs may wait to discovery due to their absence from the current transcriptomic database. A search against the KEGG annotation database showed that the majority of CCE genes were involved in the ‘Drug metabolism - other enzymes’ (ko00983) and ‘Insect hormone biosynthesis’ (ko00981) pathways.

#### Other candidate genes related to resistance

In addition, other unigenes were also identified in the *L. entomophila* transcriptome for encoding insecticide target or with a high sequence similarity to important insecticide metabolism genes. As shown in Table 2, a number of unigenes annotated as enzymes related to resistance, such as acetyl-CoA carboxylase (ACCase, 13 unigenes), superoxide dismutase (SOD, 6 unigenes), catalase (1 unigene), gamma-aminobutyric acid receptor (GABA, 9 unigenes), chloride channel (36 unigenes), and ryanodine receptors (RyRs, 20 unigenes), The average length of these unigenes was 629 bp (ACCase), 801 bp (SOD), 543 bp (GABA), and 879 bp (chloride channel). The only one unigene encoding catalase enzyme has a length of 1,573 bp. Although most of these unigenes are not fully length, they will facilitate a further characterization of these targets by RACE to retrieve the full length cDNAs.

**Figure 7:**
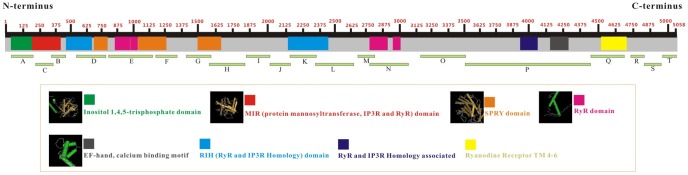
Schematic diagram of unigenes used to compile the nearly complete ryanodine receptor (RyR) amino acid sequence from *Liposcelis entomophila*. The top bold line indicates the full-length of amino acid sequence in *Pediculus humanus* (XP_002424547). The lines (A-T) represent amino acid sequence of unigenes from *L. entomophila* transcriptome. Following the order of A-T, the corresponding unigenes were: LeU18255 (537 bp), LeU18254 (324 bp), LeU18256 (331 bp), LeU5229 (709 bp), LeU28837 (1,049 bp), LeU2424 (513 bp), LeU32927 (684 bp), LeU32926 (766 bp), LeU32929 (457 bp), LeU32928 (478 bp), LeU2349 (491 bp), LeU33024 (958 bp), LeU33026 (406 bp), LeU33025 (945 bp), LeU1676 (1,065 bp), LeU3061 (2,788 bp), LeU35947 (910 bp), LeCL1197-2 (210 bp), LeCL3186-2 (299 bp), LeU35949 (389 bp).

RyRs are huge ion channels that are responsible for the release of Ca^2+^ from the sarco/endoplasmic reticulum and are the targets of two new novel classes of synthetic insecticidal chemicals, phthalic acid diamides and anthranilic diamides [55]. Recently, resistance to diamide has been reported in the diamondback moth, *P. xylostella* (Lepidoptera: Plutellidae) [56], and cDNAs encoding novel insect RyRs were cloned from the diamondback moth, the rice leaffolder, *Cnaphalocrocis medinalis* and the fruit fly, *D. melanogaster* [57]–[59]. In *L. entomophila* transcriptomic database, a total of 20 unigene sequences (with an average length of 715 bp) were identified as putative fragments of RyRs, and each of them were able to translate into amino acid sequence (Figure 7, A-T). According to the protein sequences of ryanodine receptor of the human body louse, *P. humanus* (XP_002424547), all of 20 amino acid sequences could be mapped well onto almost all of the location of the human body louse RyR, including most of RyRs domain structure (Figure 7). Our results here will facilitate cDNA cloning and provided the basis for further structural and functional characterization of *L. entomophila* RyR.

GABA receptors are prevalent in the nervous systems of insects and they are the targets of naturally occurring as well as man-made insecticides (e.g., picrotoxinin, dieldrin and fipronil). The GABA receptor consists of five subunits, each subunit containing a large extracellular agonist-binding N-terminal domain, and four transmembrane domains (M1-M4) [60]. The positions of mutations located in domains M3-M4 have been reported to associate with cyclodiene resistance in various insect species [61]. In this study, the M1, M2 and M3 regions of the GABA receptor amino acid sequences in *L. entomophila* and other insect species were aligned and it reveals the conserved sequence of amino acid residues in these regions (Figure S3). No SNPs were detected in these amino acid sequences, might due to the populations of *L. entomophila* studied here are susceptible strains or a very low level of frequency of amino acid mutations associate with insecticide resistance. For VGSC, alignments of some parts of deduced amino acid sequences from *L. entomophila* and other insect species were also conducted (domain II were not included due to the absence of this domain in our present transcriptomic database). These alignment blocks were reported with amino acid substitutions in resistant insect strains [62], [63]. Many SNPs were detected in these regions and those specificity of substitutions might potentially be associated with knockdown insecticide resistance in this pest (Figure S4). However, these mutations need to be investigated and confirmed in pyrethrins-resistant *L. entomophila*, which was selecting by laboratory selection, and will be conducted in our future researches.

### Detection of heat shock proteins

**Figure 8:**
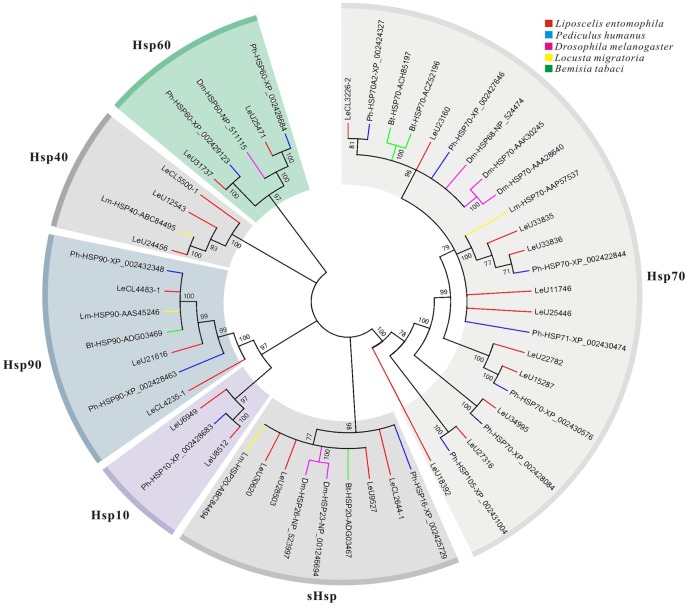
Phylogenetic analysis of putative heat shock protein (Hsp) genes in *Liposcelis entomophila* with other insects. Numbers above the branches show support for the phylogenies from amino acid sequences and only values above 70% are shown.

The heat shock proteins (Hsps) that are abundantly expressed in insects are important modulators of insect survival, and usually act as molecular chaperones, promoting correct refolding and preventing aggregation of denatured proteins [64]. They are key elements of the stress response system at the cellular level and will be up-regulated in cells exposed to a wide variety of abiotic stressors, such as heat shock, osmotic stress, and environmental contaminants (heavy metals, pesticides and polycyclic aromatic hydrocarbons), and biotic (bacteria and virus) factors [65]. Hsps represent a super gene family and can be divided into several families, including Hsp90, Hsp70, Hsp60, Hsp40, Hsp10, and small Hsps (12 to 43 kDa) based on the molecular weight (MW) and homology [66]. Recently, Hsps have been shown to increase markedly the resistance to thermal and oxidative stress in insects [67]–[69]. In this study, a total of 104 unigenes related to Hsps were identified. After manually removing short sequences, allelic variants or these have similarity with non-insect organisms, 25 putative Hsp genes remained for further analysis. Phylogenetic analysis of these Hsps revealed that the *L. entomophila* Hsps were divided into six Hsp families: Hsp90, Hsp70, Hsp60, Hsp40, Hsp10, and sHsps (Figure 8).

Hsp90 is an abundant protein under normal physiological conditions, and which play a role in contributing to the folding, maintenance of structural integrity and proper regulation of a subset of cytosolic protein [70]. In *L. entomophila* transcriptome, three Hsp90 genes were identified and all of these genes were highly expressed unigenes (reads >1,674), with an average length of 1,695 bp (Table S5). The KEGG analysis indicated that Hsp90 assigned to the pathway ‘Protein processing in endoplasmic reticulum’ (ko04141), ‘Progesterone-mediated oocyte maturation’ (ko04914), and ‘Antigen processing and presentation’ (ko04612).

A total of 11 Hsp70 genes (1,138 bp in average length) were identified from *L. entomophila* transcriptome (Table S5). Hsp70 family, the most studied in Hsps, is structurally and functionally conserved group of chaperon protein, and under stressed conditions they prevent indiscriminant protein aggregation by tightly binding denatured proteins [69]. Two of these 11 Hsp70 genes, LeU33836 and LeU33835, exhibit extremely high express level (with 287,707 and 181,722 reads, respectively) (Table S5). According to our KEGG annotation database, the majority of pathways of these Hsp70 genes were ‘Antigen processing and presentation’ (ko04612), ‘Prion diseases’ (ko05020), ‘Protein processing in endoplasmic reticulum’ (ko04141), and ‘Legionellosis’ (ko05134).

Hsp60 family is a major group of Hsps, and includes stress inducible and constitutively expressed members. The two of highly expressed Hsp60 gene LeU25471 (1,269 bp) and LeU31737 (1,633 bp) were isolated from *L. entomophila* (Table S5). These two Hsp60 genes are expressed at a high level under the normal conditions in *L. entomophila* and only LeU25471 was mapped in KEGG database with four pathways: ‘Tuberculosis’ (ko05152); ‘RNA degradation’ (ko03018); ‘Legionellosis’ (ko05134) and ‘Type I diabetes mellitus’ (ko04940).

Members of Hsp40s (also called DnaJs) have been conserved throughout evolution and are important for protein homeostasis, where they stimulate the ATPase activity of the Hsp70s that are involved in protein translation, folding, unfolding, translocation, and degradation [71]. In this study, three *L. entomophila* Hsp40 genes (with an average length of 1,003 bp) were discovered and all of them participate in the pathways ‘Influenza A’ (ko05164) and ‘Protein processing in endoplasmic reticulum’ (ko04141) (Table S5).

Hsp10 is a near 10 kDa conserved chaperone protein, which functions as a co-chaperone with Hsp60. Here, two unigenes were identified putatively encoded complete Hsp10 ORFs (LeU6949, 318 bp and LeU8512, 330 bp) (Table S5). However, both of these genes were not mapped in KEGG pathway database. Actually, the role of Hsp10 in insects has not been as clearly defined.

Small Hsps are a family of molecular chaperones, which involved in cellular defense under environmental stress conditions [64], [68]. Recently, small Hsps were extensively studied in insect, and they have been showed overexpression against environmental stress [72]–[74]. In this study, we identified four putative sHsp genes (with an average length of 533 bp) from *L. entomophila* transcriptome (Table S5). Most of them have high level of expression under the normal conditions, and KEGG analysis revealed that the major pathways for these genes were ‘Protein processing in endoplasmic reticulum’ (ko04141) and ‘VEGF signaling pathway’ (ko04370). Previous study showed higher variability and tolerance to heat stress in *L. entomophila*. The sHsp were seemed to played important role in protecting against acute thermal stress and Hsp70 family did not been detected in the same heat shock stress [14]. However, in *L. entomophila* transcriptomic database, Hsp70 genes were the major Hsp genes and all of them were mapped in KEGG database. These findings lead us to believe that Hsp70 also might be involved as chaperones providing protection against acute thermal stress in the psocid species. Therefore, further molecular studies are necessary to confirm the heat inducible proteins identified in previous study [14].

### EST-SSR and SNPs discovery

**Figure 9:**
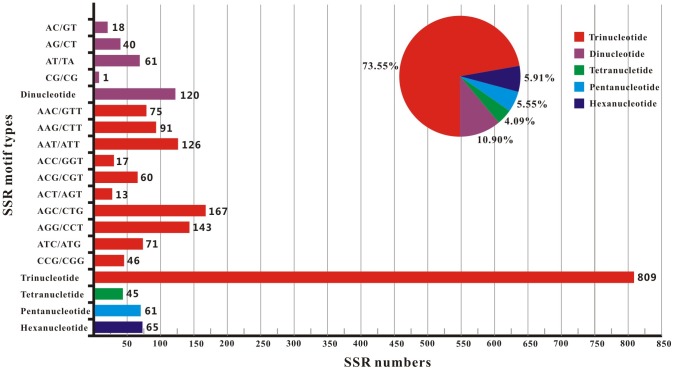
Distribution of microsatellites (SSRs) among different nucleotide types found in the transcriptome of *Liposcelis entomophila*.

SSRs are co-dominant, hyper variable, neutral and reproducible molecular markers [75], and for these reasons, they have become the most widely used molecular markers in population genetic and conservation studies, such as evaluating the level of genetic variation in a species, performing QTL analysis and constructing genetic linkage [76]. However, developing these markers through microsatellite enrichment followed by cloning and Sanger sequencing is time consuming and costly. Fortunately, the recent advent of next generation sequencing has encouraged quick and easy isolation of microsatellite markers [77]. In this study, a total of 1,100 EST-SSRs were identified and a frequency of at least one EST-SSR per 28.14 Kb in the expressed fraction of the *L. entomophila* transcriptome (Table 4). Meanwhile, most of these EST-SSRs have the number of repeats under 10 times and 5-7 times were the most abundant. Of these SSRs, the largest of fraction was trinucleotide (73.55%), followed by dinucleotide (10.90%), hexanucleotide (5.91%), pentanucleotide (5.55%), and tetranucleotide (4.09%) (Figure 9). Based on the distribution of SSR motifs, AT accounted for 50.83% of the dinucleotide repeats. In the 10 types of trinucleotide repeats, AGC (20.64%) was the most common motif, followed by AGG (17.68%) and ATT (15.57%) (Figure 9). Meanwhile, 230 SSR primer pairs were designed under stringent criteria, including 14 for dinucleotide repeats, 197 for trinucleotide repeats, 6 for pentanucleotide repeats and 13 for hexanucleotide repeats (Table S6). Currently, only a few genomic SSRs were isolated from *L. entomophila* genome in our previous study [78]. Therefore, the detection of EST-SSRs for this psocid is valuable for further study psocid intra- and inter-specific differentiation and gene flow.

**Table 4:** Summary of EST-SSRs identified in *Liposcelis entomophila* transcriptome.

SSR motifs	Number of repeats									Frequency	Mean distance
	4	5	6	7	8	9	10	>10	Total	(%)	(Kb)
Dinucleotide	–	–	67	24	15	3	6	5	120	0.22	257.94
Trinucleotide	–	487	171	128	17	0	2	4	809	1.49	38.26
Tetranucleotide	–	30	14	0	0	0	0	1	45	0.08	687.85
Pentanucleotide	50	6	2	1	1	1	0	0	61	0.11	507.43
Hexanucleotide	47	5	6	0	1	1	0	5	65	0.12	476.21
Total	97	528	260	153	34	5	8	15	1,100	2.02	28.14

Note: Frequency  =  SSR number/total number of non-redundant sequences; Mean distance  =  Total size of non-redundant sequences/total SSR number.

A total of 57,757 SNPs were identified from *L. entomophila* transcriptome database (Table 5). The predicted SNPs included 40,187 putative transitions (Ts) and 17,570 putative transversions (Tv), which giving a mean Ts: Tv ratio of 2.29∶1 across the transcriptome of *L. entomophila*. The overall frequency of all types of SNPs was one SNP per 540 bp in *L. entomophila* transcriptomic sequences. Though a large amount of putative markers (SSRs and SNPs) were acquired in this study, all of these predicted molecular markers need to be validated to rule out false positives and sequencing errors in future studies.

**Table 5:** Summary of SNPs identified from transcriptome of *Liposcelis entomophila.*

Type		Number of count	Frequency per Kb
Transition	A-G	20,229	1.53
	C-T	19,958	1.55
	Sub total	40,187	0.77
Transversion	A-C	4,431	6.99
	A-T	5,011	6.18
	C-G	3,587	8.63
	G-T	4,541	6.82
	Sub total	17,570	1.76
Total		57,757	0.54

## Conclusion

In this study, *de novo* transcriptome sequencing for the stored-product pest insect *L. entomophila* using Illumina HiSeq™ 2000 was performed for the first time. A total of 54,406,328 high-quality transcriptomic reads were obtained, giving rise to an average of 571 bp for 54,220 unigenes. A significant number of functions associated with unigenes and putative metabolic pathways were identified. Many candidate genes that are potentially involved in insecticide resistance and environmental stress, including those detoxification-related genes, target proteins of insecticides and heat shock protein were identified. In addition, the pathways associated with these candidate genes yielded new insights to better understand their functions and relations. Moreover, a large number of SSRs and SNPs were predicted and can be used for subsequent maker development and population genetic studies. All of these valuable molecular resources are worthy of further investigation. Our study provides the largest number of ESTs to database and lays the initial groundwork for in-depth, functional transcriptomic profiling of *L. entomophila*.

## Supporting Information

Figure S1 Length distribution of *Liposcelis entomophila* transcriptome sequences. A, Length distribution of unigene sequences; B, Length distribution of contig sequences.
(TIFF)

Figure S2 Distribution of *Liposcelis entomophila* unigene sequences among KEGG (Kyoto Encyclopedia of Genes and Genomes) pathways. The top 25 most highly represented pathways are shown.
(TIFF)

Figure S3 Alignment of the M1, M2 and M3 regions of the GABA receptor amino acid sequences in *Liposcelis entomophila* and other insect species reveals the conserved sequence of amino acid residues.
(DOC)

Figure S4 Alignments of some parts of deduced amino acid sequences of the voltage-sensitive sodium channel genes from *Liposcelis entomophila* and other insect species.
(DOC)

Table S1 Statistics of GO categories from *Liposcelis entomophila* transcriptomic sequences.
(DOC)

Table S2 Summary information for the manually curated P450 genes and their potentially involved in putative pathways.
(DOC)

Table S3 Summary information for the manually curated GST genes and their potentially involved in putative pathways.
(DOC)

Table S4 Summary information for the manually curated CCE genes and their potentially involved in putative pathways.
(DOC)

Table S5 Summary information for the manually curated heat shock protein genes and their potentially involved in putative pathways.
(DOC)

Table S6 Summary information for EST-SSR designed primers in *Liposcelis entomophila*.
(XLS)
