# Supplementary figures and images for: De Novo Assembly, Gene Annotation, and Marker Discovery in Stored-Product Pest Liposcelis entomophila (Enderlein) Using Transcriptome Sequences
Source: PLoS One. 2013 Nov 14;8(11):e80046. doi: 10.1371/journal.pone.0080046 (PMC3828239; doi:10.1371/journal.pone.0080046)

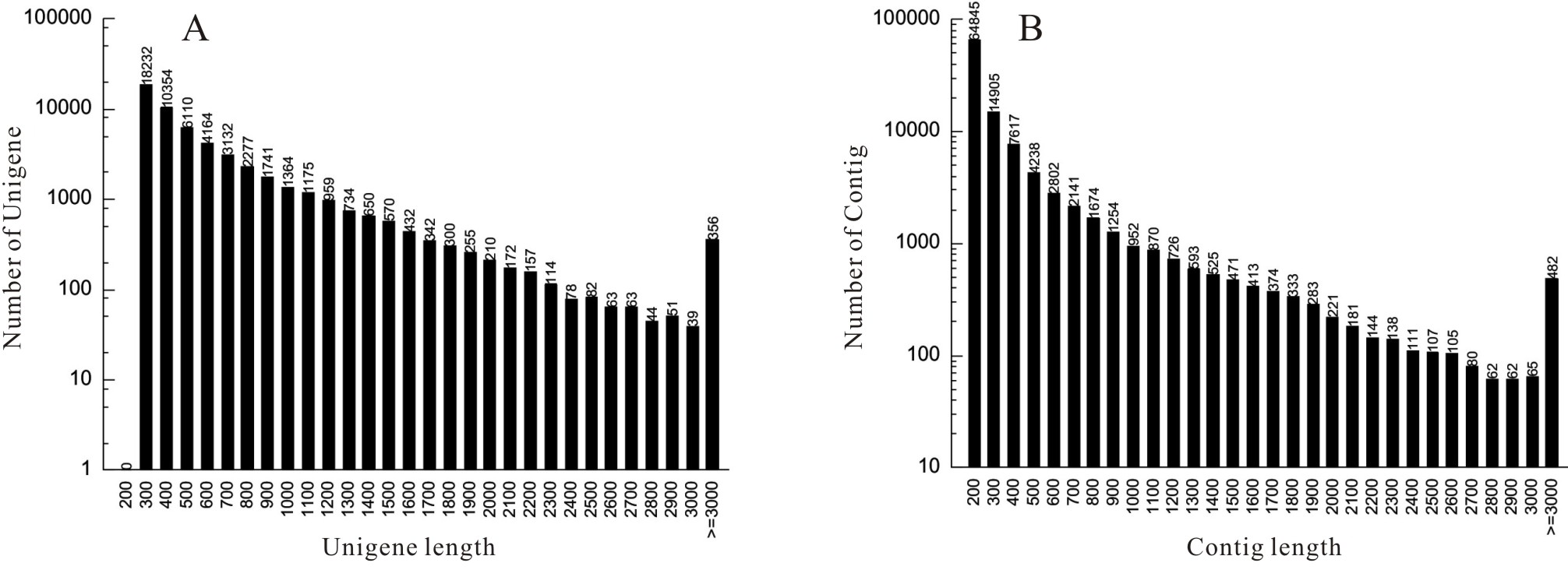

Supplement: Figure S1 — Length distribution of Liposcelis entomophila transcriptome sequences. A, Length distribution of unigene sequences; B, Length distribution of contig sequences. (TIFF) [file pone.0080046.s001.tiff]

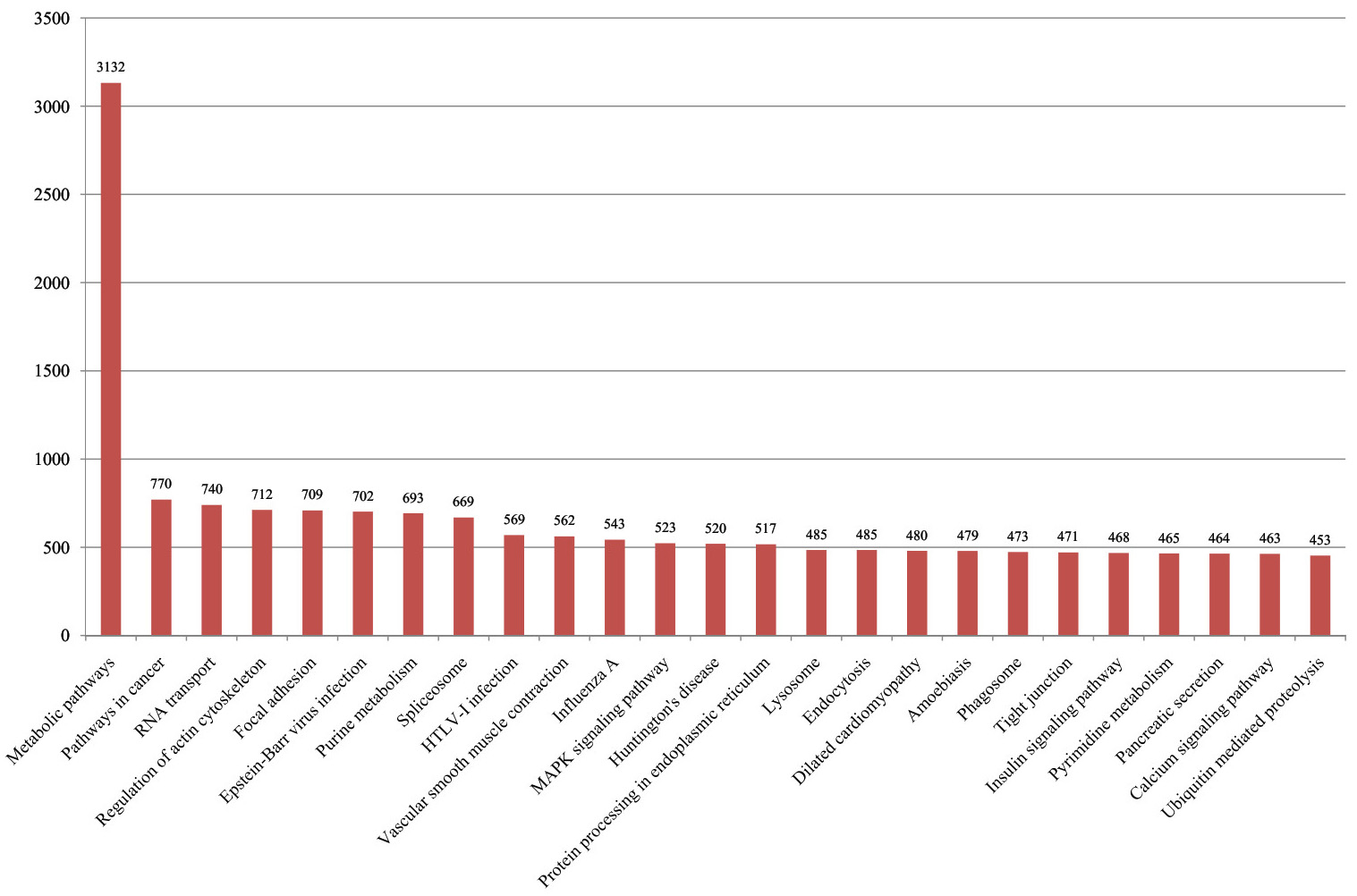

Supplement: Figure S2 — Distribution of Liposcelis entomophila unigene sequences among KEGG (Kyoto Encyclopedia of Genes and Genomes) pathways. The top 25 most highly represented pathways are shown. (TIFF) [file pone.0080046.s002.tiff]
