# Supplementary material for: De Novo Assembly, Gene Annotation, and Marker Discovery in Stored-Product Pest Liposcelis entomophila (Enderlein) Using Transcriptome Sequences
Source: PLoS One. 2013 Nov 14;8(11):e80046. doi: 10.1371/journal.pone.0080046 (PMC3828239; doi:10.1371/journal.pone.0080046)
Supplement: Figure S3 — Alignment of the M1, M2 and M3 regions of the GABA receptor amino acid sequences in Liposcelis entomophila and other insect species reveals the conserved sequence of amino acid residues. (DOC) [file pone.0080046.s003.doc]

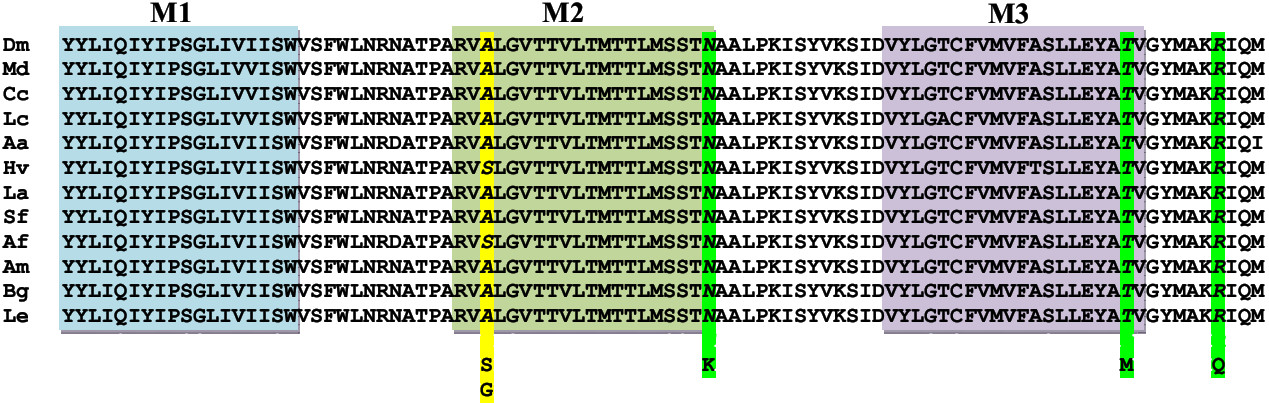


**Figure S3 Alignment of the M1, M2 and M3 regions of the GABA receptor amino acid sequences in *Liposcelis entomophila* and other insect species reveals conserved sequence of amino acid residues.**

Dm: *Drosophila melanogaster* (NP_523991); Ma: *Musca domestica* (BAD16658); Cc: *Ceratitis capitata* (AF172352); Lc: *Lucilia cuprina* (AAB81966); Aa: *Aedes aegypti* (AAA68961); Hv: *Heliothis virescens* (CAB41615); Ls: *Laodelphax striatellus* (BAF31884); Sf: *Sogatella furcifera* (BAL63029); Af: *Anopheles funestus* (ADU55585); Am: *Apis mellifera* (AAC63381); Bg: *Blattella germanica* (AAB33733); Le: *Liposcelis entomophila* (in this study). The three membrane-spanning regions (M2-M4) are marked in color box. The positions of mutations (A2’S and A2’G) have been reported to associate with cyclodiene resistance in various insect species are in yellow background, and other resistant-associated amino acid substitutions are also marked in green background which were found in specific insect species.
