# Supplementary material for: De Novo Assembly, Gene Annotation, and Marker Discovery in Stored-Product Pest Liposcelis entomophila (Enderlein) Using Transcriptome Sequences
Source: PLoS One. 2013 Nov 14;8(11):e80046. doi: 10.1371/journal.pone.0080046 (PMC3828239; doi:10.1371/journal.pone.0080046)
Supplement: Figure S4 — Alignments of some parts of deduced amino acid sequences of the voltage-sensitive sodium channel genes from Liposcelis entomophila and other insect species. (DOC) [file pone.0080046.s004.doc]

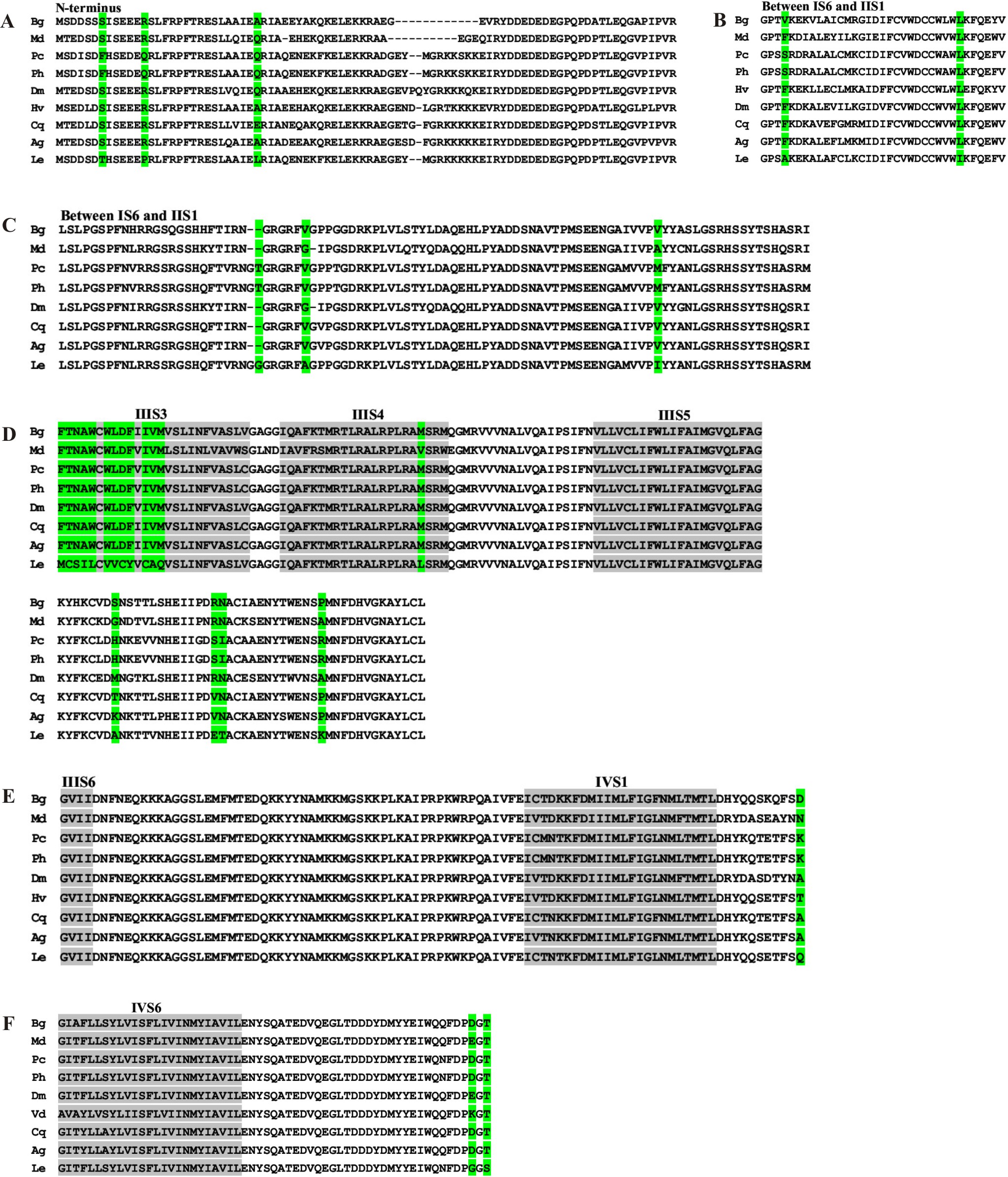


**Figure S4. Alignments of some parts of deduced amino acid sequences of the voltage-sensitive sodium channel genes from *Liposcelis entomophila* and other insect species.**

Bg, *Blattella germanica* (U73583); Md, *Musca domestica* (AAB47604); Pc, *Pediculus humanus capitis* (AAP20107); Ph, *Pediculus humanus corporis* (BAC67159); Dm, *Drosophila melanogaster* (P35500); Vd, *Varroa destructor* (AAP13992); Hv, *Heliothis virescens* (AAC26513); Cq, *Culex quinquefasciatus* (BAI77917); Ag, *Anopheles gambiae* (CAM12801); Le, *Liposcelis entomophila* (in this study). All of these alignment blocks (mutations outside of domain II) were reported with amino acid substitutions in resistant insect strains. A, The N-terminus of sodium channel; B, The region located between the membrane spanning segments IS6 and IIS1; C, The region located between the membrane spanning segments IS6 and IIS1; D, The region included domain IIIS3, IIIS4, IIIS5 and the loop connecting them; E, The region located between the membrane spanning segments IIIS6 and IVS1; F, The region included IVS6 and a part of the sequences of behind this spanning segment. The SNPs with the specific mutations to *L. entomophila* are in green background, and these regions may contain the resistant-associated amino acid substitutions.
