# Supplementary material for: De Novo Assembly, Gene Annotation, and Marker Discovery in Stored-Product Pest Liposcelis entomophila (Enderlein) Using Transcriptome Sequences
Source: PLoS One. 2013 Nov 14;8(11):e80046. doi: 10.1371/journal.pone.0080046 (PMC3828239; doi:10.1371/journal.pone.0080046)
Supplement: Table S1 — Statistics of GO categories from Liposcelis entomophila transcriptomic sequences. (DOC) [file pone.0080046.s005.doc]

**Table S1.** Statistics of GO categories from *Liposcelis entomophila* transcriptomic sequences.

| Ontology | Class | Number of unigenes | Ratio |
| --- | --- | --- | --- |
| Biological process | cellular process | 10,355 | 8.92% |
|  | metabolic process | 8,663 | 7.46% |
|  | biological regulation | 4,233 | 3.65% |
|  | single-organism process | 4,024 | 3.47% |
|  | regulation of biological process | 3,909 | 3.37% |
|  | response to stimulus | 3,294 | 2.84% |
|  | localization | 2,944 | 2.54% |
|  | establishment of localization | 2,592 | 2.23% |
|  | multicellular organismal process | 2,555 | 2.20% |
|  | signaling | 2,423 | 2.09% |
|  | developmental process | 2,302 | 1.98% |
|  | cellular component organization or biogenesis | 2,289 | 1.97% |
|  | reproduction | 851 | 0.73% |
|  | reproductive process | 835 | 0.72% |
|  | negative regulation of biological process | 755 | 0.65% |
|  | locomotion | 589 | 0.51% |
|  | positive regulation of biological process | 573 | 0.49% |
|  | biological adhesion | 475 | 0.41% |
|  | growth | 404 | 0.35% |
|  | multi-organism process | 355 | 0.31% |
|  | death | 319 | 0.27% |
|  | immune system process | 288 | 0.25% |
|  | cell proliferation | 254 | 0.22% |
|  | cell killing | 5 | 0.004% |
|  | carbon utilization | 2 | 0.002% |
| Sub total |  | 55,288 | 47.62% |
| Cellular component | cell | 8,011 | 6.90% |
|  | cell part | 8,011 | 6.90% |
|  | organelle | 4,744 | 4.09% |
|  | membrane | 3,859 | 3.32% |
|  | macromolecular complex | 2,630 | 2.27% |
|  | membrane part | 2,507 | 2.16% |
|  | organelle part | 2,434 | 2.10% |
|  | membrane-enclosed lumen | 678 | 0.58% |
|  | extracellular region | 406 | 0.35% |
|  | synapse | 286 | 0.25% |
|  | extracellular region part | 261 | 0.22% |
|  | cell junction | 251 | 0.22% |
|  | synapse part | 230 | 0.20% |
|  | extracellular matrix | 189 | 0.16% |
|  | extracellular matrix part | 76 | 0.07% |
|  | nucleoid | 7 | 0.006% |
|  | virion | 4 | 0.003% |
|  | virion part | 4 | 0.003% |
| Sub total |  | 34,588 | 29.79% |
| Molecular function | binding | 10,894 | 9.38% |
|  | catalytic activity | 10,026 | 8.64% |
|  | transporter activity | 1,355 | 1.17% |
|  | receptor activity | 1,030 | 0.89% |
|  | molecular transducer activity | 763 | 0.66% |
|  | structural molecule activity | 669 | 0.58% |
|  | enzyme regulator activity | 641 | 0.55% |
|  | nucleic acid binding transcription factor activity | 478 | 0.41% |
|  | electron carrier activity | 178 | 0.15% |
|  | antioxidant activity | 89 | 0.08% |
|  | protein binding transcription factor activity | 65 | 0.06% |
|  | channel regulator activity | 12 | 0.01% |
|  | translation regulator activity | 7 | 0.006% |
|  | nutrient reservoir activity | 5 | 0.004% |
|  | morphogen activity | 4 | 0.003% |
|  | metallochaperone activity | 2 | 0.002% |
|  | protein tag | 1 | 0.002% |
|  | receptor regulator activity | 1 | 0.002% |
| Sub total |  | 26,220 | 22.58% |
| Total |  | 116,096 | 100% |
