# Supplementary material for: De Novo Assembly, Gene Annotation, and Marker Discovery in Stored-Product Pest Liposcelis entomophila (Enderlein) Using Transcriptome Sequences
Source: PLoS One. 2013 Nov 14;8(11):e80046. doi: 10.1371/journal.pone.0080046 (PMC3828239; doi:10.1371/journal.pone.0080046)
Supplement: Table S2 — Summary information for the manually curated P450 genes and their potentially involved in putative pathways. (DOC) [file pone.0080046.s006.doc]

**Table S2.** Summary information for the manually curated P450 genes and their potentially involved in putative pathways.

| Gene name | Clade | Length (bp) | Number of reads | Putative pathways against KEGG |
| --- | --- | --- | --- | --- |
| LeU28882 | CYP2 | 1,441 | 24,479 | ko00981 |
| LeU20703 | CYP2 | 801 | 849 | ko00981 |
| LeU16133 | CYP2 | 1,458 | 3,929 | ko00981 |
| LeU25459 | CYP2 | 1,068 | 644 | ko00981 |
| LeU28073 | CYP2 | 897 | 9,526 | ko00981 |
| LeU34953 | CYP2 | 888 | 4,761 | ko00981 |
| LeU33388 | CYP2 | 1,320 | 2,170 | ko00981 |
| LeU32344 | CYP2 | 1,014 | 335 | ko00981 |
| LeU3434 | CYP2 | 1,140 | 396 | ko00981 |
| LeU34641 | Mito.Clade | 822 | 556 | ko00100 |
| LeU30516 | Mito.Clade | 1,074 | 448 | ko00981 |
| LeU29478 | Mito.Clade | 876 | 240 | ko00981 |
| LeU17229 | Mito.Clade | 1,626 | 14,685 | - |
| LeU2187 | Mito.Clade | 764 | 241 | - |
| LeU33198 | Mito.Clade | 1,107 | 327 | - |
| LeU2867 | CYP4 | 1,104 | 1,004 | - |
| LeCL689-1 | CYP4 | 798 | 175 | - |
| LeU2013 | CYP4 | 1,415 | 2,314 | - |
| LeU34330 | CYP4 | 852 | 1,554 | - |
| LeCL2499-1 | CYP4 | 990 | 448 | - |
| LeU18550 | CYP4 | 1,143 | 1,607 | - |
| LeCL5449-1 | CYP4 | 1,329 | 2,752 | - |
| LeU33937 | CYP4 | 1,518 | 3,472 | - |
| LeU23778 | CYP4 | 831 | 190 | - |
| LeCL4001-1 | CYP4 | 1,272 | 11,058 | - |
| LeCL4001-2 | CYP4 | 1,365 | 10,040 | - |
| LeU35165 | CYP4 | 1,167 | 28,774 | - |
| LeU33446 | CYP4 | 1,197 | 1,037 | - |
| LeU816 | CYP4 | 962 | 423 | - |
| LeU32122 | CYP4 | 1,437 | 4,168 | - |
| LeU28126 | CYP4 | 963 | 1,586 | - |
| LeU36246 | CYP4 | 1,500 | 3,489 | - |
| LeCL5624-1 | CYP4 | 1,053 | 1,797 | - |
| LeCL410-2 | CYP3 | 1,389 | 820 | - |
| LeCL410-3 | CYP3 | 1,404 | 722 | - |
| LeCL410-4 | CYP3 | 1,404 | 566 | - |
| LeU30992 | CYP3 | 738 | 1,981 | - |
| LeU27795 | CYP3 | 945 | 1,603 | - |
| LeCL2179-1 | CYP3 | 1,116 | 4,379 | - |
| LeCL2179-2 | CYP3 | 807 | 515 | - |
| LeU30381 | CYP3 | 849 | 3,050 | - |
| LeU31180 | CYP3 | 1,290 | 11,307 | - |
| LeCL4016-2 | CYP3 | 940 | 2,091 | - |
| LeU18998 | CYP3 | 933 | 439 | - |
| LeU28394 | CYP3 | 1,050 | 3,781 | - |
| LeU21253 | CYP3 | 1,485 | 4,780 | - |
| LeU31998 | CYP3 | 837 | 274 | - |
| Le CL2658-1 | CYP3 | 1,199 | 990 | - |
| Le CL2658-2 | CYP3 | 1,200 | 1,893 | - |
| Le CL4720 | CYP3 | 1,554 | 3,868 | - |
| LeU13178 | CYP3 | 969 | 1,321 | - |
| LeU19415 | CYP3 | 1,185 | 10,701 | - |
| LeU21565 | CYP3 | 1,241 | 1,084 | - |
| LeU29981 | CYP3 | 1,071 | 2,662 | - |
| LeU12385 | CYP3 | 842 | 546 | - |
| LeU22015 | CYP3 | 996 | 1,971 | - |
| LeCL1352-1 | CYP3 | 834 | 219 | - |
| LeU17618 | CYP3 | 1,237 | 4,189 | ko04976; ko00983; ko00982; ko00980; ko00830; ko00140; ko00591 |
| LeU3146 | CYP3 | 1,407 | 3,317 | - |
| LeU33830 | CYP3 | 1,518 | 11,329 | - |
| LeCL5675-1 | CYP3 | 786 | 7,063 | - |
| LeCL5675-2 | CYP3 | 945 | 5,220 | - |
| LeCL1981-1 | CYP3 | 1,530 | 2,443 | - |
| LeCL1981-2 | CYP3 | 1,353 | 2,247 | - |
| LeU1257 | CYP3 | 810 | 517 | - |
| LeU34047 | CYP3 | 1,386 | 3,419 | - |
| LeU27069 | CYP3 | 831 | 255 | - |
| LeU31362 | CYP3 | 1,170 | 3,671 | ko04976; ko00983; ko00982; ko00980; ko00830; ko00140; ko00591 |

Pathway names: ko00981 Insect hormone biosynthesis; ko00100 Steroid biosynthesis; ko04976 Bile secretion; ko00983 Drug metabolism - other enzymes; ko00982 Drug metabolism - cytochrome P450; ko00980 Metabolism of xenobiotics by cytochrome P450; ko00830 Retinol metabolism; ko00140 Steroid hormone biosynthesis; ko00591 Linoleic acid metabolism.

KEGG, the Kyoto Encyclopedia of Genes and Genomes pathway database.

The dash ‘-’, no map in KEGG database.
