# Supplementary material for: De Novo Assembly, Gene Annotation, and Marker Discovery in Stored-Product Pest Liposcelis entomophila (Enderlein) Using Transcriptome Sequences
Source: PLoS One. 2013 Nov 14;8(11):e80046. doi: 10.1371/journal.pone.0080046 (PMC3828239; doi:10.1371/journal.pone.0080046)
Supplement: Table S3 — Summary information for the manually curated GST genes and their potentially involved in putative pathways. (DOC) [file pone.0080046.s007.doc]

**Table S3.** Summary information for the manually curated GST genes and their potentially involved in putative pathways.

| Gene name | Class | Length (bp) | Number of reads | Putative pathways against KEGG |
| --- | --- | --- | --- | --- |
| LeU21814 | Delta | 396 | 1,903 | ko05215; ko00480; ko00982; ko00980 |
| LeU6016 | Delta | 657 | 1,233 | ko05215; ko00480; ko00982; ko00980 |
| LeU4219 | Delta | 489 | 102 | ko05215; ko00480; ko00982; ko00980 |
| LeU6150 | Delta | 648 | 19,856 | ko05215; ko00480; ko00982; ko00980 |
| LeU11873 | Delta | 465 | 378 | ko05215; ko00480; ko00982; ko00980; ko05200 |
| LeU37151 | Delta | 642 | 133 | ko05215; ko00480; ko00982; ko00980 |
| LeU6272 | Delta | 450 | 1,647 | ko05215; ko00480; ko00982; ko00980 |
| LeU36161 | Delta | 654 | 4,207 | ko05215; ko00480; ko00982; ko00980 |
| LeU18464-1 | Delta | 612 | 2,306 | ko05215; ko00480; ko00982; ko00980; ko05200 |
| LeU18464-2 | Delta | 618 | 2,306 | ko05215; ko00480; ko00982; ko00980; ko05200 |
| LeCL1114-1 | Delta | 552 | 1,313 | ko05215; ko00480; ko00982; ko00980; ko05200 |
| LeU6126 | Delta | 411 | 3,674 | ko05215; ko00480; ko00982; ko00980 |
| LeU36144 | Delta | 651 | 9,995 | ko05215; ko00480; ko00982; ko00980 |
| LeU11767 | Delta | 597 | 52,570 | ko05215; ko00480; ko00982; ko00980; ko05200 |
| LeU6314 | Delta | 642 | 18,799 | ko05215; ko00480; ko00982; ko00980 |
| LeCL2561-1 | Delta | 468 | 644 | ko05215; ko00480; ko00982; ko00980; ko05200 |
| LeCL2561-2 | Delta | 446 | 571 | ko05215; ko00480; ko00982; ko00980; ko05200 |
| LeU4001 | Theta | 459 | 78 | ko05215; ko00480; ko00982; ko00980 |
| LeU21773 | Theta | 609 | 407 | ko05215; ko00480; ko00982; ko00980 |
| LeU31744 | Theta | 678 | 4,911 | ko05215; ko00480; ko00982; ko00980 |
| LeU14761 | Omega | 738 | 2,710 | ko05215; ko00480; ko00982; ko00980; ko05200 |
| LeU29712 | Zeta | 381 | 579 | ko00350 |
| LeU1680 | Sigma | 648 | 834 | ko05215; ko00480; ko00982; ko00980; ko05200 |
| LeU21246 | Sigma | 633 | 445 | ko05215; ko00480; ko00982; ko00980; ko05200; ko00590 |
| LeU36414 | Sigma | 603 | 825 | ko00590 |
| LeU36282 | Sigma | 573 | 1,495 | ko05215; ko00480; ko00982; ko00980; ko00590 |
| LeU12069 | Sigma | 558 | 71 | ko00590 |
| LeU6295 | Sigma | 579 | 1,745 | ko05215; ko00480; ko00982; ko00980; ko00590 |
| LeU22058 | Sigma | 615 | 31,9195 | ko05215; ko00480; ko00982; ko00980 |
| LeU833 | Sigma | 615 | 388 | ko05215; ko00480; ko00982; ko00980; ko00590 |
| LeU11087 | Sigma | 609 | 1,008 | ko05215; ko00480; ko00982; ko00980; ko05200; ko00590 |
| LeCL3550-3 | Sigma | 257 | 163 | ko05215; ko00480; ko00982; ko00980; ko05200; ko00590 |
| LeU21975 | Sigma | 381 | 2,106 | ko05215; ko00480; ko00982; ko00980; ko00590 |
| LeU21974 | Sigma | 454 | 2,578 | ko05215; ko00480; ko00982; ko00980; ko00590 |
| LeCL1214-4 | Sigma | 618 | 5,232 | ko05215; ko00480; ko00982; ko00980; ko05200; ko00590; ko01100 |
| LeU18298 | Microsomal | 519 | 92 | - |
| LeU6308 | Microsomal | 456 | 8,302 | ko05215; ko00480; ko00982; ko00980 |

Pathway names: ko05215 Prostate cancer; ko00480 Glutathione metabolism; ko00982 Drug metabolism - cytochrome P450; ko00980 Metabolism of xenobiotics by cytochrome P450; ko05200 Pathways in cancer; ko00350 Tyrosine metabolism; ko00590 Arachidonic acid metabolism; ko01100 Metabolic pathways.

KEGG, the Kyoto Encyclopedia of Genes and Genomes pathway database.

The dash ‘-’, no map in KEGG database.
