# Supplementary material for: De Novo Assembly, Gene Annotation, and Marker Discovery in Stored-Product Pest Liposcelis entomophila (Enderlein) Using Transcriptome Sequences
Source: PLoS One. 2013 Nov 14;8(11):e80046. doi: 10.1371/journal.pone.0080046 (PMC3828239; doi:10.1371/journal.pone.0080046)
Supplement: Table S4 — Summary information for the manually curated CCE genes and their potentially involved in putative pathways. (DOC) [file pone.0080046.s008.doc]

**Table S4.** Summary information for the manually curated CCE genes and their potentially involved in putative pathways.

| Gene name | Clade | Length (bp) | Number of reads | Putative pathways against KEGG |
| --- | --- | --- | --- | --- |
| LeCL2154.C1 | A | 1,536 | 2,900 | ko01100; ko04972; ko00983; ko00561; ko04975; ko00981; ko00100 |
| LeU34706 | A | 1,680 | 3,043 | ko04972; ko00983; ko00561; ko04975; ko00100 |
| LeCL1683.C2 | A | 1,447 | 1,808 | ko01100; ko04972; ko00561; ko04975; ko00981; ko00100 |
| LeU36096 | A | 1,605 | 78,519 | ko04972; ko00983; ko00561; ko04975; ko00100 |
| LeCL1480.C1 | A | 1,234 | 528 | ko01100; ko04972; ko00983; ko00561; ko04975; ko00100 |
| LeU14443 | F | 1,710 | 4,158 | ko00981; |
| LeU26637 | E | 1,173 | 559 | ko01100; ko00983; ko00981 |
| LeCL2903.C1 | E | 1,149 | 1,472 | ko01100; ko00983; ko00981 |
| LeCL4137.C1 | E | 1,122 | 1,648 | ko00983; ko00981 |
| LeU34088 | D | 1,116 | 2,240 | ko04972; ko00983; ko00561; ko04975; ko00981; ko00100 |
| LeCL465.C1 | D | 1,134 | 192 | ko00983; ko04514; ko00981 |
| LeU35602 | D | 1,143 | 1,289 | ko00983; ko00981 |
| LeU31367 | D | 1,134 | 1,583 | ko00983; ko00981 |
| LeCL1745.C1 | D | 1,626 | 11,450 | ko00983; ko00981 |
| LeU24755 | H | 1,398 | 342 | ko00564; ko04725; ko04514; |
| LeU593 | H | 1,737 | 1,472 | ko00564; ko04725; ko04514 |
| LeCL2507.C1 | H | 1,686 | 1,459 | ko01100; ko00983; ko04514 |
| LeCL5795.C1 | J | 1,572 | 1,153 | ko00564; ko04725 |
| LeU6950 | J | 1,914 | 663 | ko00564; ko04725 |

Pathway names: ko01100 Metabolic pathways; ko04972 Pancreatic secretion; ko00983 Drug metabolism - other enzymes; ko00561 Glycerolipid metabolism; ko04975 Fat digestion and absorption; ko00981 Insect hormone biosynthesis; ko00100 Steroid biosynthesis; ko04514 Cell adhesion molecules (CAMs); ko00564 Glycerophospholipid metabolism; ko04725 Cholinergic synapse.

KEGG, the Kyoto Encyclopedia of Genes and Genomes pathway database; The dash ‘-’, no map in KEGG database.
