# Supplementary material for: De Novo Assembly, Gene Annotation, and Marker Discovery in Stored-Product Pest Liposcelis entomophila (Enderlein) Using Transcriptome Sequences
Source: PLoS One. 2013 Nov 14;8(11):e80046. doi: 10.1371/journal.pone.0080046 (PMC3828239; doi:10.1371/journal.pone.0080046)
Supplement: Table S5 — Summary information for the manually curated heat shock protein genes and their potentially involved in putative pathways. (DOC) [file pone.0080046.s009.doc]

**Table S5.** Summary information for the manually curated Heat shock protein genes and their potentially involved in putative pathways.

| Gene name | Family | Length (bp) | Number of reads | Putative pathways against KEGG |
| --- | --- | --- | --- | --- |
| LeCL4483-1 | Hsp90 | 2,175 | 62,686 | ko05200; ko04141; ko05215; ko04914; ko04612; ko04621 |
| LeU21616 | Hsp90 | 1,092 | 1,993 | ko04141; ko05215; ko04914; ko04612; ko04621 |
| LeCL4235-1 | Hsp90 | 1,818 | 1,868 | - |
| LeU11746 | Hsp70 | 1,233 | 2,134 | ko05169; ko03040; ko05164; ko04010; ko04141; ko04144; ko05145; ko05162; ko05020; ko05134; ko04612 |
| LeU25446 | Hsp70 | 859 | 513 | ko03040; ko05164; ko04010; ko04141; ko04144; ko05145; ko05162; ko05020; ko05134; ko04612 |
| LeU22782 | Hsp70 | 960 | 350 | ko04141; ko05020; ko04612; ko03060 |
| LeU15287 | Hsp70 | 1,386 | 32,474 | ko04141; ko05020; ko04612; ko03060 |
| LeU34995 | Hsp70 | 1,332 | 8,487 | ko05152; ko03018 |
| LeU27316 | Hsp70 | 2,100 | 7,405 | ko04141 |
| LeU18392 | Hsp70 | 837 | 1,351 | ko05169; ko03040; ko05164; ko04010; ko04141; ko04144; ko05145; ko05162; ko05020; ko05134; ko04612; ko03060 |
| LeU33836 | Hsp70 | 720 | 287,707 | ko05145; ko05162; ko05020; ko05134; ko04612 |
| LeU33835 | Hsp70 | 1,017 | 181,722 | ko05145; ko05162; ko05020; ko05134; ko04612 |
| LeU23160 | Hsp70 | 990 | 175 | ko05169; ko03040; ko05164; ko04010; ko04141; ko04144; ko05145; ko05162; ko05020; ko05134; ko04612 |
| LeCL3226-2 | Hsp70 | 1,089 | 3,219 | ko05169; ko03040; ko05164; ko04010; ko04141; ko04144; ko05145; ko05162; ko05020; ko05134; ko04612 |
| LeU25471 | Hsp60 | 1,269 | 9,821 | ko05152; ko03018; ko05134; ko04940 |
| LeU31737 | Hsp60 | 1,633 | 9,514 | - |
| LeCL5500-1 | Hsp40 | 1,212 | 3,955 | ko04141 |
| LeU12543 | Hsp40 | 735 | 181 | ko05164; ko04141 |
| LeU24456 | Hsp40 | 1,062 | 1,867 | ko05164; ko04141 |
| LeU6949 | Hsp10 | 318 | 213 | - |
| LeU8512 | Hsp10 | 330 | 1,085 | - |
| LeU30620 | sHsp | 464 | 9,269 | ko04141 |
| LeU28503 | sHsp | 594 | 7,017 | ko04141 |
| LeU9527 | sHsp | 516 | 114 | ko04370 |
| LeCL2644-1 | sHsp | 558 | 4,845 | ko05169; ko04010; ko04141; ko05146; ko04370 |

Pathway names: ko05200 Pathways in cancer; ko04141 Protein processing in endoplasmic reticulum; ko05215 Prostate cancer; ko04914 Progesterone-mediated oocyte maturation; ko04612 Antigen processing and presentation; ko04621 NOD-like receptor signaling pathway; ko05169 Epstein-Barr virus infection; ko03040 Spliceosome; ko05164 Influenza A; ko04010 MAPK signaling pathway; ko04144 Endocytosis; ko05145 Toxoplasmosis; ko05162 Measles; ko05020 Prion diseases; ko05134 Legionellosis; ko03060 Protein export; ko05152 Tuberculosis; ko03018 RNA degradation; ko04940 Type I diabetes mellitus; ko04370 VEGF signaling pathway; ko05146 Amoebiasis.

KEGG, the Kyoto Encyclopedia of Genes and Genomes pathway database; the dash ‘-’ means no map in KEGG database.
